# Scale-Mark-Based Gauge Reading for Gauge Sensors in Real Environments with Light and Perspective Distortions

**DOI:** 10.3390/s22197490

**Published:** 2022-10-02

**Authors:** Chia-Hui Wang, Ke-Kai Huang, Ray-I Chang, Chien-Kang Huang

**Affiliations:** 1 Department of Computer Science and Information Engineering, Ming Chuan University, No. 5, De Ming Road, Taoyuan 33348, Taiwan; 2 Department of Engineering Science and Ocean Engineering, National Taiwan University, No. 1, Sec. 4, Roosevelt Road, Taipei 10617, Taiwan

**Keywords:** principal components analysis (PCA), optical character recognition (OCR), gauge center detection, gauge reading

## Abstract

Nowadays, many old analog gauges still require the use of manual gauge reading. It is a time-consuming, expensive, and error-prone process. A cost-effective solution for automatic gauge reading has become a very important research topic. Traditionally, different types of gauges have their own specific methods for gauge reading. This paper presents a systematized solution called SGR (Scale-mark-based Gauge Reading) to automatically read gauge values from different types of gauges. Since most gauges have scale marks (circular or in an arc), our SGR algorithm utilizes PCA (principal components analysis) to find the primary eigenvector of each scale mark. The intersection of these eigenvectors is extracted as the gauge center to ascertain the scale marks. Then, the endpoint of the gauge pointer is found to calculate the corresponding angles to the gauge’s center. Using OCR (optical character recognition), the corresponding dial values can be extracted to match with their scale marks. Finally, the gauge reading value is obtained by using the linear interpolation of these angles. Our experiments use four videos in real environments with light and perspective distortions. The gauges in the video are first detected by YOLOv4 and the detected regions are clipped as the input images. The obtained results show that SGR can automatically and successfully read gauge values. The average error of SGR is nearly 0.1% for the normal environment. When the environment becomes abnormal with respect to light and perspective distortions, the average error of SGR is still less than 0.5%.

## 1. Introduction

**Figure 1:**
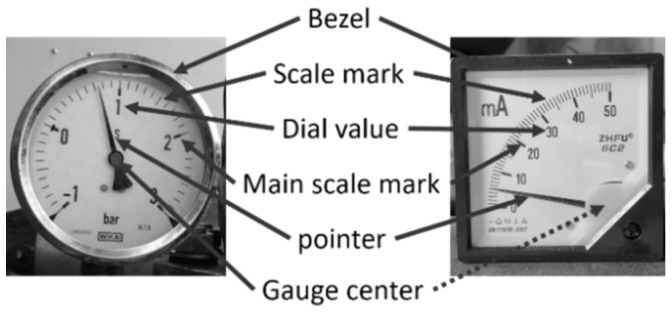
Structures of a circular gauge and an arc gauge.

Industrial automation is an unstoppable trend because of its profitable benefits in industrial evolution. However, many old analog gauges still exist in different sectors. Those who engage in manual gauge reading must spend time travelling to the gauges to read them and write down their current values. It is a time-consuming, expensive, and error-prone process. Thus, automatic gauge reading has become a very important research topic. It uses a camera to capture images of the analog gauge to cost-effectively read out the gauge values via computer vision techniques. Although analog gauges may have different shapes of their bezels, there are usually two categories of gauges in their charts: circular and arc. As shown in Figure 1, they have five visible characteristics: a bezel, scale mark, pointer, dial value, and gauge center.

Traditionally, there have been three main categories of research on gauge reading. In [1,2,3,4,5,6], the gauge center was extracted, and then the angle between the pointer and the gauge center was calculated. Based on the angle, the gauge value can be determined using interpolation technology. In [7,8], the spatial domain information was converted to the polar domain with the gauge center as the transform center; thus, the scale marks have been converted from a circular shape to a linear one. Linear interpolation can also be used to calculate gauge values. In [9,10,11], the gauge value was determined by deep learning neural networks. For all the above research, the determination of the gauge center is the most critical process.

To ascertain the gauge center, [1,2] employed the circular feature of the bezel for a circle-fitting algorithm. Consequently, the center of the bezel was the gauge center. In [3,4], the authors used the light circle feature on the pointer to find the gauge center. The center of the light circle was determined to be the gauge center. In [5], the authors used the fact that the background of a gauge usually has a specific color to find the gauge center. The center of the area with the specific color was defined as the gauge center. The authors of [6] used the center positions of the dial values to ascertain the gauge center using a circle-fitting algorithm since these center positions have the characteristics of co-circularity. In [7,8], the authors used scale marks to obtain the gauge center. The authors of [7] used the extended lines of the scale marks and applied their intersection to find the gauge center. The authors of [8] used the centroid of the scale marks and main scale marks with co-circularity characteristics to find the gauge center. In [9,10,11], the authors use deep learning neural networks to obtain the gauge center.

**Figure 2:**
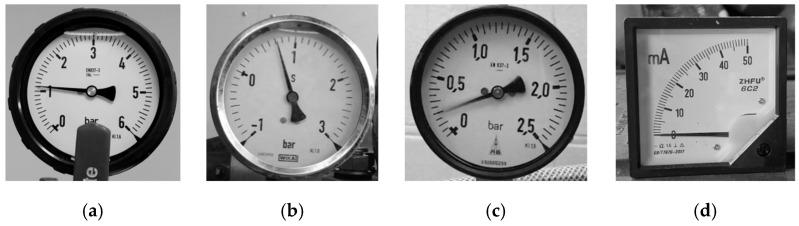
Four common gauges. (**a**) Circular gauge. (**b**) Circular Gauge with negative dial value. (**c**) Circular Gauge with floating dial value. (**d**) Arc gauge.

Figure 2 shows four common analog gauges. Based on shape of the gauge chart, Figure 2a–c are circular gauges. Figure 2d is an arc gauge. Note that, in Figure 2a, there is a shelter in the circular bezel to disturb the round characteristic of the bezel. In this paper, the shape of the bezel is unnecessary to be round or square. Note that the gauge center usually appears in the central area of the circular gauge. On the contrary, it appears in the bottom right area on the arc gauge, as shown in Figure 2d. Traditionally, the circular and arc gauges have their own specific methods for gauge reading, but the generality of these methods for different-type gauges is insufficient. Deep learning neural networks may be applicable to all types of gauges, but it is not easy to train deep neural networks with all types of gauges. Their performance may be poor. This paper presents a systematized solution to automatically read the values for both analog circular and arc gauges.

Since most gauges have scale marks (circular or in an arc), a new gauge-reading algorithm called SGR (Scale-mark-based Gauge Reading) is proposed. The main idea of SGR is to regard the scale marks as connected components (CCs) and use said scale marks to find the gauge center. Three features in CCs (the area, compactness, and ratio of CCs) are used to extract the scale marks. The eigenvectors corresponding to the scale marks are extracted by principal components analysis (PCA). Since the primary eigenvectors of scale marks point to the gauge center, the intersection of these extended vectors can be founded to be the gauge center. In this paper, SGR mainly operates on the spatial domain. The main scale marks are obtained by the characteristic of area and angle of scale marks. The dial values are extracted by an open optical character recognition (OCR) tool called Keras-OCR [12]. The main scale marks and the dial values are bound together if their angles are the same. The gauge value is obtained by a new interpolation method that can work successfully even if one of the right bindings or left bindings are lost. Since Keras-OCR is not specifically designed for the values on the gauges, it will mistakenly determine the dial values with a negative or floating number. SGR uses the common difference feature to automatically correct the error values of Keras-OCR.

**Figure 3:**
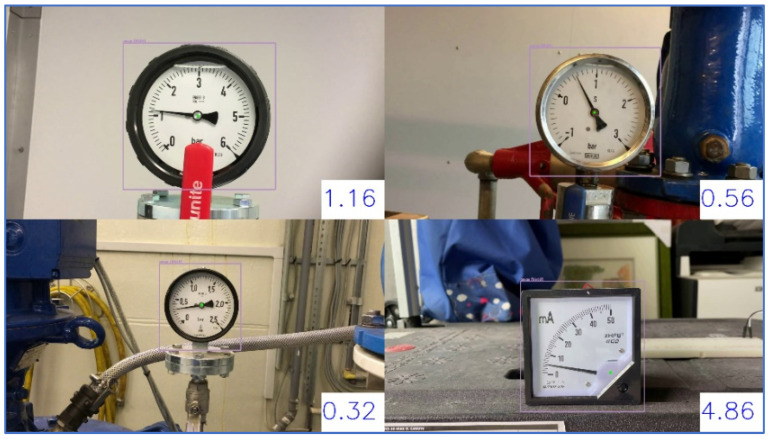
Snapshot of our SGR experiments in real environments.

As shown in Figure 3, our experiments use four videos in real environments with light and perspective distortions to test different gauge-reading methods. Video 1, Video 2, and Video 3 are taken from Internet. Video 4 is a video produced by us. The gauges in the video are first detected by YOLOv4 (You only look once) [13] and the detected regions are clipped as the input images for SGR. The obtained results show that SGR can successfully read the gauge values. The average errors are nearly 0.1% for the normal environment. When the environment becomes abnormal due to the light and perspective distortions, the average errors are still less than 0.5%. The rest of this paper is organized as follows: Section 2 describes related works; Section 3 presents the SGR method; then, the experimental results are shown in the next section; and finally, the conclusion and future works are given in Section 5.

## 2. Related Works

In 2015, Chi et al. used computer vision to detect the gauge value [5]. They assumed that the center of the background was the gauge center. The background usually has a specific color (a white color in [5]); therefore, the highest pixel value in the image is automatically detected as the seed. Based on the seed, a region-growing scheme is then used to obtain the background. The center of this background is set to be the gauge center. Then, this image in the spatial domain is transformed to the polar domain by the found gauge center as its circle center. The region of the scale marks is extracted by the feature of the fixed black and white point scale. This region uses Otsu’s method to obtain the binarization image. This image calculates its angle histogram, and the larger values are the main scale marks. Two lines of the pointer bezel are detected using the Hough transform method. The intersection of these lines is the pointer peak. Then, a directed line from the gauge center to this pointer peak can be generated as the pointer. Finally, the gauge value is calculated by an interpolation between the angle of the main scale marks and pointer. The biggest problem with this method is that the reflection of light will influence the detection of the white background. Therefore, the gauge center is incorrect, resulting in the wrong gauge value.

In 2016, Zheng et al. used computer vision to detect an arc gauge’s value [8]. They used Multi-Scale Retinex with Color Restoration (MSRCR) [14] to reduce the effect of the light source. The calibration of the gauge image is performed by perspective transform. The binarized image is then obtained using the adaptive threshold [15]. The scale marks are extracted by the Hough transform. These scale marks have the characteristics of co-circularity. Therefore, the gauge center is obtained by the circle Hough transform (CHT) method. The pointer and the pointer’s angle are extracted by a thinning algorithm [16] and Hough transform, respectively. The image is then converted to a polar domain and uses the connected-component-labeling (CCL) [17] method to obtain the minimum and maximum scale mark positions. Finally, the gauge value is obtained by interpolation.

In 2017, Selvathai et al. analyzed the values of a vehicle gauge [2]. They used Canny edge detection to extract its bezel. Then, CHT was utilized to find the center, which is denoted as the gauge center. The gauge pointer is the longest line and then can be determined by the Hough transform. The gauge value is obtained by interpolation between the pointer peak and the gauge center. The biggest problem of the method in [2] is that an ideal circular bezel is difficult to obtain.

In 2017, Yifan et al. proposed a specific solution for finding the gauge center [3]. This solution assumed that there is a light circle in the pointer and the center of the light circle is identified as the gauge center. Then, it captured a rectangular area around the center of the image that would include the light circle. The pointer is the largest CC in this region of interest (ROI). A round-degree mechanism is then employed to fit the circle. The obtained center is the gauge center. Considering this gauge center as circle center, the CC of the pointer is then transformed to the polar domain after using the thinning method and Hough transform. The pointer peak is extracted by the largest polar angle in the polar angle frequency histogram. Then, an improved least squares method is applied to obtain the pointer. It assumed that the zero-scale line appears in the lower left corner of the gauge center. It uses the polar radius frequency histogram to remove the arc line. The largest CC uses the least squares error method to find the corresponding straight-line equation. This line is a zero-scale line. Finally, the gauge value is obtained by interpolation between the angle of the pointer and its line. The biggest problem of [3]’s method is that the light circle does not appear in each gauge. Thus, it cannot apply for all kinds of gauges and the zero-scale line is not always shown on the bottom left of the gauge.

In 2019, Lauridsen et al. used image processing to a read circular gauge [1]. In their method, it is necessary to first remove any noise. Then, use the characteristics of the round bezel and the least squares-fitting method to obtain the gauge center. Then, use the five features to identify the scale marks and the pointer using K-means clustering. The angle of the pointer is obtained by PCA. The experimental result has a good performance in their test videos. However, the biggest problem of [1]’s solution is that an ideal circular bezel is difficult to be obtain. Since the appearance of the bezel is diverse and as this diversity will interfere with circle fitting, the obtained gauge center will be incorrect. In the arc gauges, there are no round bezels to ascertain the gauge center.

In 2019, Sheng et al. used a double Hough space voting scheme to ascertain the gauge center [7]. It transfers the image of the gauge from the spatial domain to Hough space via the Hough transform twice. The scale mark lines can be obtained by the first vote of the Hough transform. The gauge center is then extracted by the second vote. In [6], it was mentioned that the performance of the double Hough space voting algorithm is not good because zigzag lines would appear when the scale marks were not vertical or horizontal. The corresponding line equations of the scale marks are deviated, resulting in the inaccurate determination of the gauge center.

In 2020, Li et al. proposed a technique [6] to automatically read gauges based on text detection. It uses a Fast Oriented Text-Spotting (FOTS) [18] neural network to extract the position and number of dial values. These positions can calibrate the gauge image. The gauge center is obtained by least squares circle fitting because the positions of the dial value have a co-circularity characteristic based on the gauge center. The image is transformed to the polar domain. The locations of the dial values are obtained by using the bounding box that can include all positions of dial values. Above this region of bounded box, there is another region with the same area size, and this region is set as the first region of interest (ROI). The pointer is obtained by the vertical projection method, whereby the maximum value is the pointer. Based on the pointer, the dial values closest to the pointer to the left and right are then found. These dial values’ bounding boxes are set as the second ROI. The main scale marks are obtained by the vertical projection method. Finally, the gauge value is obtained by interpolation.

In 2020, Cai et al. used deep learning technology to read a gauge’s value [10]. This technique converts the gauge-reading problem into image classification and image regression problems. For each gauge, many gauge images with different effects can be produced automatically. This operation is called Virtual Sample Generation (VSG). Each value is treated as a category and as an input to a convolutional neural network (CNN) for training. This network can classify the gauge and ascertain the corresponding gauge value, but the error of this value is large. Therefore, an additional CNN network is needed for regression after the previous network finishes the classification. The result is the gauge value. This method uses a variety of techniques for the data’s augmentation, such as cropping, rotation, flipping, occlusion, etc. However, they did not illustrate how to generate a large number of virtual samples and they reported that this CNN regression network is difficult to train.

In 2020, Meng et al. used deep learning technology to detect gauge-related parameters [9], the gauge center, and the positions of the minimum and maximum scale marks. In contrast to the study of [10] (which predicted gauge values directly), this solution used the heatmap method to represent the above three parameters. Each parameter has a corresponding position on the heatmap. These parameters are predicted by the Hourglass network [19]. This would generate a virtual pointer from the gauge center to the maximum scale mark. This virtual pointer is a mask, and it scans all the value positions from small to large. During the virtual pointer’s rotational scan on the binarized gauge image, the numbers of founded black points on this pointer mask are calculated. The angle of the black point with the highest number is the angle of the pointer. The limitation of [9]’s method is that it has to input the dial values of the minimum and maximum manually. The gauge value is then calculated by the angle of the pointer and these dial values.

In 2021, Howells et al. applied deep learning technology to detect the gauge value by using smartphones [11]. It uses the CenterNet network [20] to detect gauge-related parameters. Different from the study of [9], only the gauge center and the positions of the minimum and maximum scale marks were found. This network will output four positions. In addition to the above-mentioned positions of the three points, the pointer is also predicted. These positions are also predicted by a heatmap, which is the same as in [9]. The limitation of [11]’s method is that it has to input dial values manually too. The gauge value is obtained by interpolation. Since the pointer’s position will appear unstable for different the gauges applied, the gauge-reading performance is worse than in [9].

## 3. SGR (Scale-Mark-Based Gauge Reading) Algorithm

**Figure 4:**
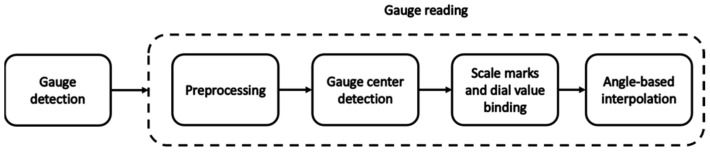
Diagram of SGR architecture.

Figure 4 shows the SGR architecture diagram that is divided into two parts. The first part entails gauge detection. The second part entails gauge reading with four phases, including preprocessing, gauge center detection, binding (between scale marks and dial values), and angle-based interpolation.

SGR uses YOLOv4 to detect gauges. YOLOv4 [13] is a commonly used neural network algorithm for object detection. It uses Mosaic’s data augmentation from the input side. Its architecture is mainly divided into three parts: a backbone, neck and head. The backbone is used to extract features. The neck is used to receive and combine the features obtained from the backbone. The head will predict and classify the objects in the image based on these characteristics.

SGR is similar to the current surveillance systems in which cameras are fixed to the front of the gauges perpendicularly and read the gauge values continuously. For detecting gauges with YOLOv4, two or more perpendicularly fixed images are uploaded for system initialization. LabelImg [21], a type of labeling tool, is then employed to label the uploaded images. Different data augmentation technologies including different image distortions and lighting effects are utilized to increment the YOLOv4 dataset.

**Figure 5:**
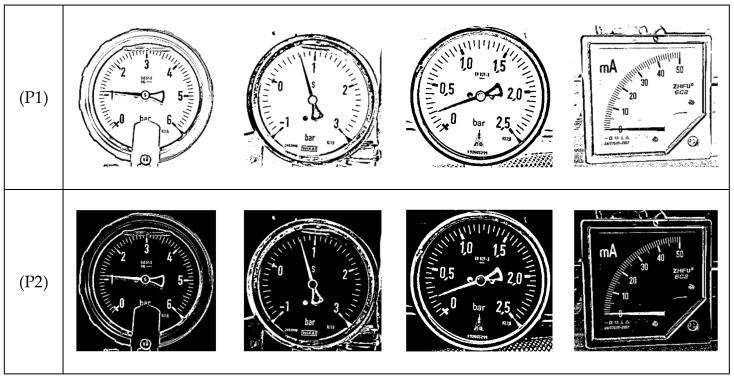

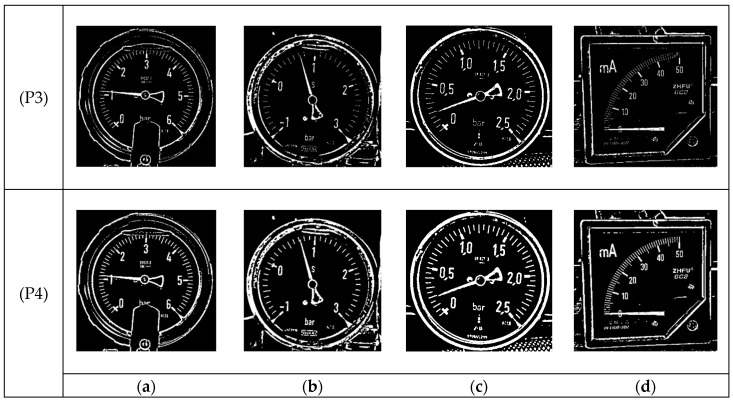
Results of preprocessing. (**a**) Circular gauge. (**b**) Circular Gauge with negative dial value. (**c**) Circular Gauge with floating dial value. (**d**) Arc gauge.

In SGR, the color gauge image is first converted into a grayscale image (as Figure 2). Then, the adaptive threshold is used to binarize the image (as Figure 5 (P1)). Since scale marks, dial values, and pointers are black on the gauge, these objects have inverted image color with respect to the main objects in the CC (as Figure 5 (P2)). Erosion (as Figure 5 (P3)) and dilation (as Figure 5 (P4)) are then employed to remove noise.

### 3.1. Gauge Center Detection

SGR can be applied to both circular and arc gauges. In order to extract the gauge center, SGR uses the characteristic wherein the primary eigenvectors of the scale marks will intersect at the gauge center. There are many objects in a gauge, such as scale marks, dial values, and a pointer. These objects will form CCs independently. The CCs belonging to scale marks have many characteristics. Their corresponding areas are similar, their corresponding locations are adjacent, etc. The proposed algorithm uses these features to first find the CCs of the scale marks. PCA is then employed to find the eigenvectors of these CCs. Finally, the intersection of these primary eigenvectors is obtained as the gauge center. There are three steps: the CCs’ detection, the scale marks’ detection, and the gauge center’s detection.

*CCs’ detection*: The *findContours* function of OpenCV is applied to find all the CCs in the gauge region. This function uses the connecting characteristics of the boundaries in the binary image to determine the outer border and hole border and their hierarchical relationships. In this way, each boundary can be represented by a CC [22]. For all the CCs, each component can find the corresponding rotated bounding box. Let an area variable of *a* be the number of pixels in the component. Let *w* and *h* be the width and the height of the rotated bounding box, respectively. The proposed method uses the CC’s compactness *c* and ratio *r^wh^* to find the scale marks. The calculation formula of *c* and *r^wh^* is as follows.

(1) $$\{\begin{array}{c} c=a/\left(w\times h\right)\\ r^{wh}=w/h \end{array}$$

*Scale marks’ detection*: Most of the scale marks can be divided into two types: main and minor. The main scale marks represent important information in a gauge, and they point to the dial value, but they are limited in number. The minor scale marks are designed to increase the precision between the main scale marks. The number of the minor scale marks is greater than that of the main scale marks. Therefore, the proposed method focuses on the minor scale marks. The lengths of the scale mark lines with the same types are the same in the image. Therefore, they can be utilized to select the same scale mark lines. We use three steps to obtain minor scale marks. The first step is to find possible short scale marks (i.e., a minor scale mark) by compactness *c*. Since short scale marks are usually short straight lines, their corresponding compactness *c* is limited to 1. We need to set a tolerance range from *TH*1 to *TH*2 of the compactness values to detect the minor scale marks in the CCs. This value in the range is greater than the threshold value *TH*1, and its value is less than the threshold value *TH*2. The proposed method’s *TH*1 and *TH*2 are 0.8 and 1.2, respectively. The second step is to calculate the largest *r^wh^* of the CCs obtained in the first step. The results are then fed into the third step. The third step computes the majority of the CCs’ areas obtained in the second step. The CCs corresponding to this area *a* are the scale marks that have been found. It is not necessary to detect all the minor scale marks in our proposed scheme. However, if the undetected minor scale marks are too numerous to detect the gauge center, an error signal will be generated as follows: “gauge center detection”.

**Figure 6:**
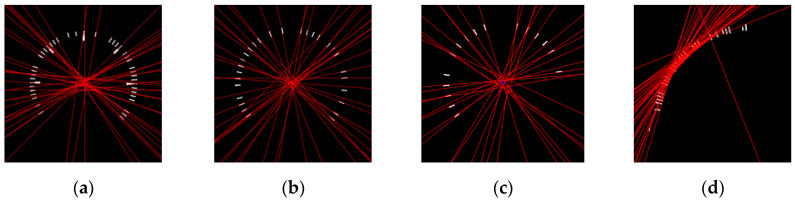
Results of Double Hough space voting. (**a**) Circular gauge. (**b**) Circular Gauge with negative dial value. (**c**) Circular Gauge with floating dial value. (**d**) Arc gauge.

**Figure 7:**
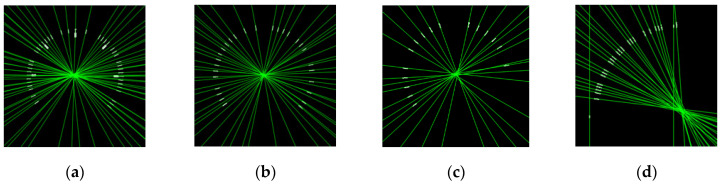
Results of the primary eigenvectors obtained by the PCA technique. (**a**) Circular gauge. (**b**) Circular Gauge with negative dial value. (**c**) Circular Gauge with floating dial value. (**d**) Arc gauge.

*Gauge center detection*: When the minor scale marks are obtained, each minor scale mark can be considered as a line segment. The corresponding extended lines will intersect at the gauge center. Double Hough space voting [7] utilizes this characteristic to extract the gauge center. Zigzag lines will appear in Double Hough space voting. Figure 6 shows the result of the applied Double Hough space voting. The effect of the deviation can be easily seen. It is hard to extract the gauge center using Double Hough space voting. In SGR, each minor scale mark is considered as a CC. The corresponding eigenvectors of minor scale marks are extracted by PCA. Figure 7 shows the extracted eigenvectors, where the primary eigenvectors of the minor scale marks intersect at the gauge center. This paper uses this characteristic to obtain the gauge center. The following describes the basis of the PCA. When the primary eigenvectors of the minor scale marks are obtained, every two linear equations have an intersection point. The majority numbers of linear equations for the intersection points are all computed. When the number of scale marks corresponding to this majority is greater than half of the number of short scale marks, this majority is set as the gauge center. Otherwise, SGR will generate the “Type I Error” message.

### 3.2. Scale Marks and Dial Values Binding

The main purpose of SGR is to establish the relationship between the main scale marks and dial values. Thus, there is no need to manually enter the dial values, and so the automation of gauge reading for circular and arc gauges can be achieved. This subsection has three steps: the detection of the main scale marks, the detection and inference of the dial values, and the binding of the main scale marks and dial values.

#### 3.2.1. Detection of Main Scale Marks

We used the short scale marks to find the gauge center in the previous subsection. However, the main scale marks have not yet been found. Now, we introduce an algorithm to detect the main scale marks. The algorithm is divided into two steps: find the candidates of the main scale marks, and then extract and infer the main scale marks based on these candidates.

This proposed algorithm first calculates the distances from the gauge center to the upper and lower boundaries of the short scale marks. They are denoted as *d*1 and *d*2, respectively. *d*1 *−* *d*2 can be considered the length of a short scale mark. In general, the main scale marks are longer than the short scale marks and the extra length usually occurs near the gauge center. The proposed algorithm assumes that the length of a main scale mark is double that of a short scale mark. Therefore, the proposed algorithm extracts a ring with a radius ranging from *d*1 to 2 × *d*2 − *d*1 based on the gauge center.

**Figure 8:**
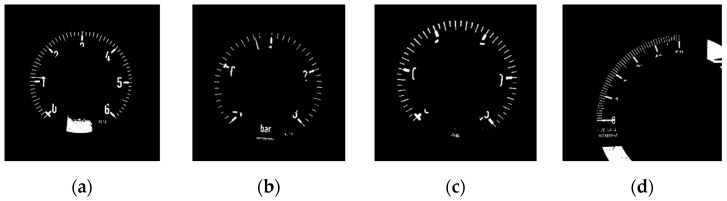
Binarized results of the possible area of main scale marks. (**a**) Circular gauge. (**b**) Circular Gauge with negative dial value. (**c**) Circular Gauge with floating dial value. (**d**) Arc gauge.

**Figure 9:**
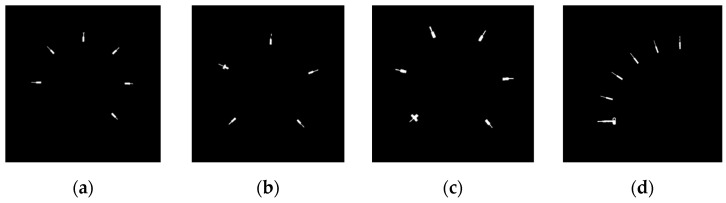
Results of MSMCs. (**a**) Circular gauge. (**b**) Circular Gauge with negative dial value. (**c**) Circular Gauge with floating dial value. (**d**) Arc gauge.

After acquiring the possible region of main scale marks, the proposed algorithm first binarizes the region with a threshold value of *TH*3, where *TH*3 is the average gray-level value of the short scale marks obtained in the previous subsection. The CCs in the binarized image are then extracted (as shown in Figure 8). Since the area of the main scale marks is usually larger than that of the short scale marks and the first eigenvector of main scale marks will point to the gauge center, the elements in the CCs become candidates for the main scale marks if they meet the above criteria. For the criterion in using area to find candidates of main scale marks, the threshold value *TH*4 is set to be 1.5 times the average area of the short scale marks. The main scale mark candidates obtained by the above scheme are denoted MSMC and Figure 9 shows the MSMC obtained from Figure 8. In this paper, the set of these main scale mark candidates are abbreviated as MSMC.

**Figure 10:**
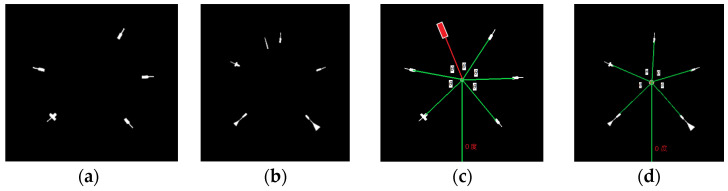
Examples of wrong results of the main scale mark detection. (**a**) Circular gauge. (**b**) Circular Gauge with negative dial value. (**c**) Circular Gauge with floating dial value. (**d**) Arc gauge.

In Figure 9, most of the main scale marks can be correctly identified from the founded MSMC, except one main scale mark in the lower left corner of Figure 9a. There are still some main scale marks that could not be extracted from the MSMC. Figure 9b and Figure 10a shows some examples. There is a missing main scale mark in Figure 10a and there is an additional main scale mark in Figure 10b. The additional main scale mark is the result of the pointer overlapping with a short scale mark. Thus, another method is presented to solve this problem, as follows.

The angles formed by the main scale mark candidates and the gauge center are calculated and sorted in ascending order. The differences between two successive angles are then obtained. The majority number of these angle differences can be found and then the angle difference in majority is the major angle difference among the main scale marks. This major angle difference is denoted as θ in this paper.

Let MSM be the set of main scale marks that will be obtained by the proposed method. When θ is obtained, the proposed method checks the angle difference between two successive members of the MSMC to determine whether it is equal to θ or not. If the answer is yes, the above two main scale mark candidates are regarded as the main scale marks. They are then appended to the MSM. Only part of the main scale marks can be obtained by the above process. The missed main scale marks can also be inferred by θ. The proposed method checks the angle difference between two successive members of the MSM to determine whether it is larger than θ. If the answer is yes, the angle difference would be divided by θ. Suppose the divided result is *n*; then, *n* − 1 main scale marks would be embedded in the above two successive main scale marks. Figure 10 provides the obtained MSM. An embedded main scale mark represented with a red rectangle is shown in Figure 10a and a misjudged main scale mark is removed in Figure 10b.

#### 3.2.2. Detection and Inference of Dial Values

**Figure 11:**
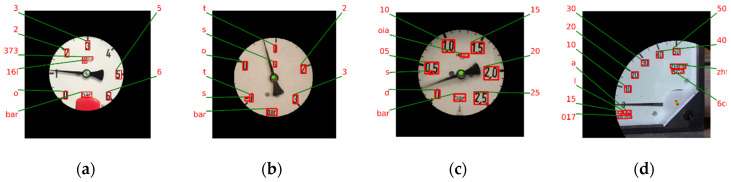
Results of Keras-OCR. (**a**) Circular gauge. (**b**) Circular Gauge with negative dial value. (**c**) Circular Gauge with floating dial value. (**d**) Arc gauge.

For the detection of the dial values, a straightforward method is to use Keras-OCR [23]. Let the strings detected by Keras-OCR be the set $W^{str}$ and the corresponding regions be the set $W^{reg}$. Some scale marks would interfere with Keras-OCR’s recognition. We overcome this problem using a scheme to remove scale marks before applying Keras-OCR. This scheme involves extracting a circle with a radius of *d*2 based on the gauge center for Keras-OCR to detect the dial values. Figure 11a–d show the $W^{str}$ and $W^{reg}$ of the proposed scheme. Keras-OCR can detect most of the dial values, but some specific numbers can be misjudged to be some letters. For example, ‘0’, ‘1’, and ‘2’ would be misjudged to be ‘o’, ‘t’, and ‘z’, respectively. The proposed algorithm corrects this problem using key-to-value mapping, where the keys are the above specific letters, and the values are the corresponding numbers. This proposed algorithm then replaces the letters of $W^{str}$ with corresponding numbers. Consequently, all the elements of $W^{str}$ would be numerical. However, there are still some problems reading gauges with negative dial values and floating dial values since Keras-OCR is not designed for a gauge’s dial values.

When Keras-OCR encounters negative dial values, the minus sign cannot be recognized correctly. Figure 11b shows that the ‘−1’ dial value detected by Keras-OCR becomes a ‘1’ whose minus sign is lost. In order to overcome this problem, the proposed algorithm uses the ‘0’ obtained by Keras-OCR to further inference the negative numbers. The members of $W^{str}$ are sorted in a clockwise order according to their angles. For members of $W^{str}$ located before element ‘0’, they can be judged as negative and a minus sign can be applied to them.

Moreover, when Keras-OCR encounters floating dial values, the dot cannot be detected in most cases. Figure 11c shows that the ‘0.5’ dial value detected by Keras-OCR becomes ‘05’, for which the corresponding dot is lost. The inference scheme is introduced below. For elements in $W^{str}$ whose length is larger than one, the connected components of the corresponding $W^{reg}$ are extracted. If the number of the connected components is equal to the length of the element plus one, the element is inferred as a floating number. In Figure 11c, the length of ‘05’ is two and the number of connected components of the corresponding region in $W^{reg}$ is three. This satisfies the above criteria. Therefore, ‘05’ is inferred to be ‘0.5’. Let DV be the set of final results of the dial value detection and inference algorithm. Figure 11 shows the DV of Figure 11a–d inferred by SGR.

#### 3.2.3. Binding of Main Scale Marks and Dial Values

This subsection focuses on the issues of binding between the main scale marks and dial values. After the binding process, every main scale mark has a corresponding dial value. The main idea is to use the angles of MSM and DV. For every main scale mark in the MSM, a dial value in DV can be binding to it if their angles are approximately equal. Most of the main scale marks have corresponding dial values now, but some of the main scale marks may not.

**Figure 12:**
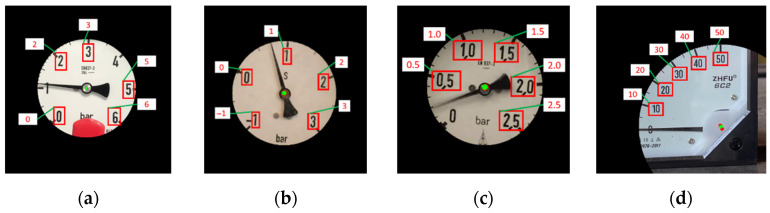
Results of dial value inference. (**a**) Circular gauge. (**b**) Circular Gauge with negative dial value. (**c**) Circular Gauge with floating dial value. (**d**) Arc gauge.

**Figure 13:**
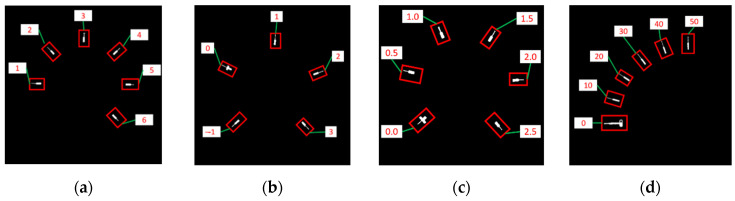
Results of the main scale mark and dial value binding. (**a**) Circular gauge. (**b**) Circular Gauge with negative dial value. (**c**) Circular Gauge with floating dial value. (**d**) Arc gauge.

To ensure that the proposed algorithm works correctly, there must be at least three successful bindings between the main scale marks and dial values. This is a constraint of this proposed algorithm. If three successful bindings cannot be generated, SGR will generate a “Type II Error” message. The process to compensate for the lost dial values is described below. For every two successive main scale marks, the compensated scheme computes the dial value difference per *θ*. The majority of the above values are calculated and set to be the value gap denoted as α in this paper. Then, lost dial values can be inferred by α and the above successful bindings. The final binding results between the main scale marks in Figure 9 and dial values in Figure 12 are shown in Figure 13.

### 3.3. Angle-Based Gauge-Reading Algorithm

**Figure 14:**
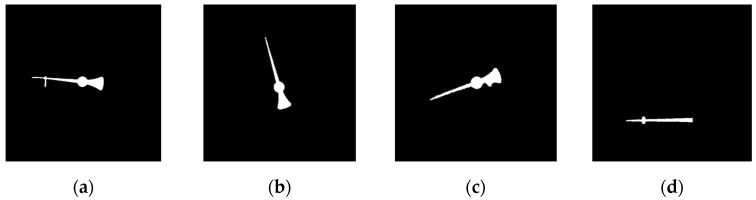
Results of pointers extracted by SGR. (**a**) Circular gauge. (**b**) Circular Gauge with negative dial value. (**c**) Circular Gauge with floating dial value. (**d**) Arc gauge.

This subsection focuses on the issue of gauge reading with respect to the gauge pointer. This proposed gauge-reading scheme is divided into two steps: the detection of the pointer and the use of the linear interpolation of the angles to obtain the gauge value. To read the gauge value, the position of the pointer needs to be detected. In a gauge, the CC formed by the pointer is usually the largest, and this CC’s primary eigenvector will point to gauge center. The appearance of the pointer is thin and long. The pointer is also closest to the gauge center. The proposed scheme uses these characteristics to obtain the pointer. Figure 14 shows the pointer extracted by the proposed scheme for Figure 5. The convex hull of the pointer is first extracted. The distances between the points in the convex hull and the gauge center are then calculated. The point with the longest distance is denoted as the pointer peak. Let the angle of this pointer peak be $\theta_{pointer}$.

As mentioned regarding the binding of the main scale marks and dial values in the previous subsection, the proposed algorithm must have at least three successful bindings between the main scale marks and dial values to work correctly. Suppose the angles of the successful bindings range from $\theta_{min}$ to $\theta_{max}$. The gauge value can be interpolated in the following three cases. For the first case where $\theta_{pointer}$ is between $\theta_{min}$ and $\theta_{max}$, the left nearest and the right nearest main scale marks for the pointer peak are extracted first. Let the corresponding angles of the extracted main scale marks be $\theta_{l}$ and $\theta_{r}$ and their corresponding dial values be $V_{l}$ and $V_{r}$. The gauge value $V_{pointer}$ is calculated by Equation (2).

(2) $$V_{pointer}=V_{l}+\frac{\theta_{pointer}-\theta_{l}}{\theta_{r}-\theta_{l}}\times \left(V_{r}-V_{l}\right)$$

For the second case where $\theta_{pointer}$ is smaller than $\theta_{min}$, the right nearest two main scale marks for the pointer peak are extracted. Let the corresponding angles of the extracted main scale marks be $\theta_{r1}$ and $\theta_{r2}$($\theta_{r2}>\theta_{r1}$) and their corresponding dial values be $V_{r1}$ and $V_{r2}$. The gauge value $V_{pointer}$ is calculated by Equation (3)

(3) $$V_{pointer}=V_{r1}+\frac{\theta_{pointer}-\theta_{r1}}{\theta_{r2}-\theta_{r1}}\times \left(V_{r2}-V_{r1}\right)$$

For the third case where $\theta_{pointer}$ is larger than $\theta_{max}$, the left nearest two main scale marks for the pointer peak are extracted. Let the corresponding angles of the extracted main scale marks be $\theta_{l1}$ and $\theta_{l2}$($\theta_{l2}>\theta_{l1}$) and their corresponding dial values be $V_{l1}$ and $V_{l2}$. The gauge value $V_{pointer}$ is calculated by Equation (4).

(4) $$V_{pointer}=V_{l2}+\frac{\theta_{pointer}-\theta_{l2}}{\theta_{l2}-\theta_{l1}}\times \left(V_{l2}-V_{l1}\right)$$

The values of Figure 2a–d are 1.11, 0.72, 0.31, and 0.57, respectively. SGR can correctly obtain these gauge values.

### 3.4. Processing of Abnormal Environments

Applications for the automatic gauge reading method are divided into two scenarios. The first involves a camera that is set on a robot that automatically moves to find gauges and extract gauge values. The second is similar to the current surveillance systems in which cameras, perpendicularly fixed in front of the gauges, read the gauge values continuously. The proposed SGR belongs to the second category.

Due to human factors or natural factors (such as earthquakes), the perpendicular gauge images captured by the camera cannot always be guaranteed. There are three possible image deformation cases: image horizontal/vertical movements, image rotation, and image perspective distortion.

YOLOv4 can overcome the problem of the image horizontal/vertical movements since YOLOv4 can detect objects in different locations. In addition, the proposed SGR is not affected by the problem of image rotation, since the image rotation will not destroy the structure of the scale marks, and the gauge value is extracted by angle interpolation.

**Figure 15:**
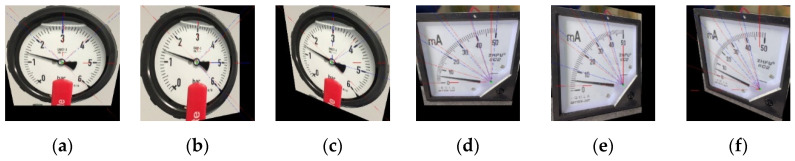
Results of image perspective distortion. (**a**) X-axis rotated 30 degrees on circular gauge. (**b**) Y-axis rotated 30 degrees on circular gauge. (**c**) X-axis rotated 30 degrees and Y-axis rotated −30 degrees on circular gauge. (**d**) X-axis rotated 30 degrees on arc gauge. (**e**) Y-axis rotated 30 degrees on arc gauge. (**f**) X-axis rotated 30 degrees and Y-axis rotated −30 degrees on arc gauge.

Finally, we discuss the problems caused by image perspective distortion. When image perspective distortion occurs, the outlines of the scale marks are obscured. Figure 15a–c show a circular gauge deformed by a rotation of 30 degrees on an X axis, Y axis, and X-Y axis, respectively. Figure 15d–f show an arc gauge deformed by a rotation of 30 degrees on X axis, Y axis and X-Y axis, respectively. The above deformation is so serious that SGR will generate an alarm.

A scheme to detect the image perspective distortion is presented as follows. For every gauge, SGR utilizes the two main scale marks to read the value. When the angle between the utilized two main scale marks is not almost equal to *θ* and the error exceeds a given threshold TH, it indicates that the profiles of the scale marks are obscured. TH is the threshold to specify the sensitivity to the image perspective distortion. The methods of detecting image perspective distortion can be divided into the following three cases. When the angle interpolation method uses Equation (2), an alarm message will be generated with Equation (5)

(5) $$\left(\theta_{r}-\theta_{l}\right)/\theta >TH$$

When the angle interpolation method uses Equation (3), an alarm message will generate with Equation (6)

(6) $$\left(\theta_{r2}-\theta_{r1}\right)/\theta >TH$$

When the angle interpolation method uses Equation (4), an alarm message will generate with Equation (7)

(7) $$\left(\theta_{l2}-\theta_{l1}\right)/\theta >TH$$

Another abnormal environmental condition occurs when light changes. The proposed algorithm uses Multi-Scale Retinex with color restoration (MSRCR), an image enhancement technique, to handle the light change problem. In order to reduce the computational complexity of SGR, MSRCR will not be enabled for every gauge image. MSRCR is only launched when SGR generates a Type I or Type II error, and then SGR is processed again to read the MSRCR-enabled gauge image.

## 4. Experimental Results

**Figure 16:**
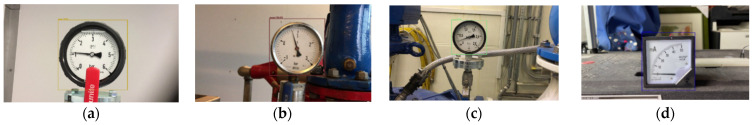
Gauges detected by YOLOv4. (**a**) Video 1. (**b**) Video 2. (**c**) Video 3. (**d**) Video 4.

This paper uses a PC as the development and testing platform, and Python and OpenCV are used as the development tools. There are four test videos in our experiments. Video 1, Video 2, and Video 3 are videos with a circular gauge taken from the internet and Video 4 is a video with an arc gauge taken by this study’s authors. Figure 16 shows the gauges detected by YOLOv4. The detected regions of the gauges are clipped as the input images in our experiments.

### 4.1. The Performance of SGR

In the above four videos, each video captures three clip images as the test data. The ground truth of each gauge center is obtained by manual marking. Let the position of the manual gauge center be ($x_{m}$, $y_{m}$) and the gauge center obtained by the test algorithm be ($x_{a}$, $y_{a}$). The ground truth of each gauge value is obtained by manual marking. Let the manually labeled gauge value be $v_{m}$, the gauge value obtained by the test algorithm be $v_{a}$, and the range from the minimum dial value to the maximum dial value be $v_{range}$. The error of the test algorithm can be calculated by Equation (8).

(8) $$\left(\frac{\left|v_{a}-v_{m}\right|}{v_{range}}\right)\times 100\%$$

In Table 1, the experimental results show that the average reading-value error of [3,4] is 0.79%. However, SGR has the best performance with an error of 0.13%. The reason is that the angle range of the interpolation in [3,4] is from the angle of the main scale mark with the minimum dial value to the main scale mark with the maximum dial value. However, SGR constructs bindings of main scale marks and dial values. A smaller interpolation error can be obtained by a smaller angle range. The performance of SGR is better than that of these previous methods.

### 4.2. Performance Evaluations of Abnormal Environments

This paragraph discusses the influence of image perspective distortion on SGR. Regarding the perspective distortion effect on the circular gauge with an X-axis rotation with various angles ranging from −30 degrees to 30 degrees and *TH* = 0.04, the perspective distortion can be tolerated by the SGR method for the rotation angle between −15 degrees and 15 degrees. The error is less than 1.5%. When the rotation angle is greater than 15 degrees and less than −15 degrees, the SGR method will generate an alarm message. When *TH* = 0.06, SGR can tolerate a relatively larger perspective distortion for the rotation angle between −21 degrees and 21 degrees. When the rotation angle is between −21 degrees to 21 degrees, the error is less than 2%. This indicates a larger *TH*, a larger tolerance, and a larger error. The error can be less than 0.5% when the rotation angle is between −10 degrees to 10 degrees.

When the rotation axis is the Y-axis, the performance of SGR is similar to that of the above. For *TH* = 0.06, the rotation from −24 degrees to 24 degrees can be tolerated. The error is less than 2%. When the X-axis and Y-axis rotate at the same time, the tolerable rotation angle is reduced, in which the range is from −12 degrees to 12 degrees. Once again, the error is less than 2%.

Regarding the perspective distortion effects on the arc gauge by rotation cases of X-axis only, Y-axis only, and X-axis and Y-axis at the same time, their tolerable rotation angle range is smaller than that of the circular gauges. When *TH* = 0.06, the tolerable rotation angle ranges from −12 degrees to 12 degrees for X-axis or Y-axis rotations. The error is less than 2%. The error can be less than 0.5% when the rotation angle is between −6 degrees to 6 degrees.

However, when the X-axis and Y-axis rotate at the same time, the tolerated rotation angle (from −9 degrees to 9 degrees) is reduced. This phenomenon occurs because the rotation center of the arc gauge is located at the bottom right corner of the gauge, while the rotation center of the perspective distortion is located at the center of the gauge.

**Figure 17:**
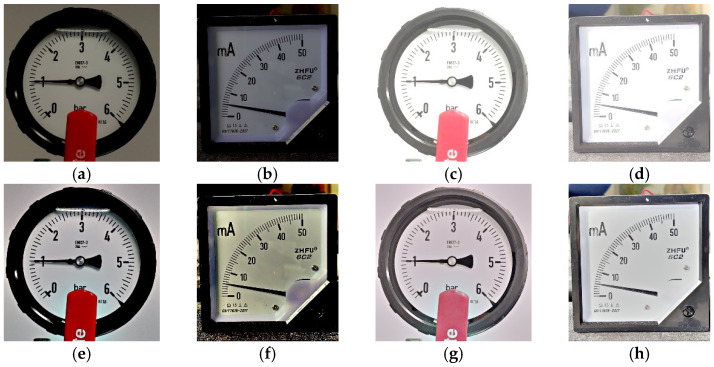
Test images in dark conditions and with bright light sources. (**a**) Original image; beta = −100. (**b**) Original image; beta = −100. (**c**) Original image; beta = 75. (**d**) Original image; beta = 75. (**e**) MSRCR result; beta = −100. (**f**) MSRCR result; beta = −100. (**g**) MSRCR result; beta = 75. (**h**) MSRCR result; beta = 75.

**Figure 18:**
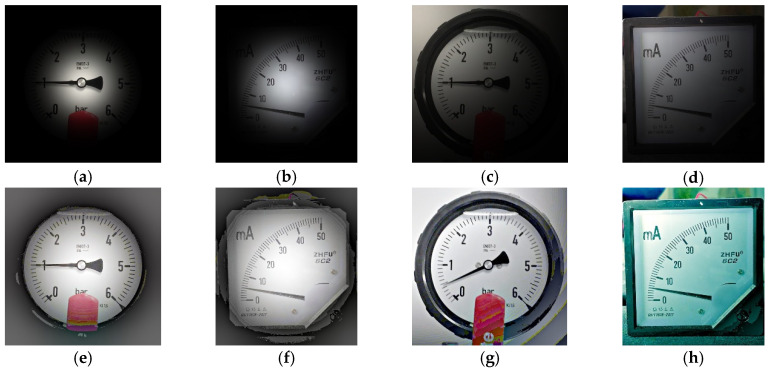
Test images of a single light source illuminating different positions on the gauge. (**a**) Original image; sigma = 100. (**b**) Original image; sigma = 100. (**c**) Original image; sigma = 300. (**d**) Original image; sigma = 300. (**e**) MSRCR result; sigma = 100. (**f**) MSRCR result; sigma = 100. (**g**) MSRCR result; sigma = 300. (**h**) MSRCR result; sigma = 300.

The influence of different light changes on SGR is illustrated as follows. Table 2 shows the results of SGR with and without MSRCR for bright and dark environments in different light changes. There are some cases generating alarms for the original SGR algorithm. However, their gauge values can be extracted when MSRCR is utilized. Next, the influence of a single light source illuminating different locations on a gauge is explored. Table 3 provides the corresponding results. There are 45 cases generating alarms for the original SGR algorithm. However, their gauge values are extracted when MSRCR is utilized. SGR demonstrates the improved performance in this environment. Figure 17 and Figure 18 show some examples of the above experiment.

## 5. Conclusions and Future Works

This paper mainly proposes a general automatic gauge-reading algorithm. This algorithm can obtain the central position of circular gauges and arc gauges using PCA. Consequently, the main scale marks and dial values will be extracted and then bound together. Our method has resolved the problem involving dial values that are negative or floating point. Therefore, it does not require manually inputting relevant parameters and further automates the system. The experimental results demonstrate that the proposed algorithm has good performance in automatic gauge reading for both circular and arc gauges. In the future, we plan to conduct more experiments on different types of gauges to detect even more information such as the measuring units appearing in the gauges and to verify the performance of the proposed algorithm.

## Figures and Tables

**Table 1:** Average errors of gauge reading (N.A. is Not Available for the applied methods).

Clip/Method	Video 1			Video 2			Video 3			Video 4			Average Error
	1-1	1-2	1-3	2-1	2-2	2-3	3-1	3-2	3-3	4-1	4-2	4-3	
[1,2]	1.17%	1.33%	0.67%	0.25%	0.75%	3.50%	0.80%	0.80%	1.20%	N.A.	N.A.	N.A.	1.16%
[3,4]	0.17%	0.33%	0.33%	0.00%	1.25%	4.25%	0.40%	0.40%	0.00%	N.A.	N.A.	N.A.	0.79%
[5]	0.33%	0.67%	0.17%	0.75%	0.75%	3.25%	0.40%	0.40%	0.00%	N.A.	N.A.	N.A.	0.75%
[6]	1.00%	0.67%	1.17%	0.75%	3.50%	0.75%	0.80%	1.20%	1.20%	0.34%	0.64%	0.28%	1.02%
[7]	0.17%	0.17%	0.50%	0.25%	1.50%	4.25%	0.40%	0.40%	0.80%	N.A.	N.A.	N.A.	0.94%
[8]	0.83%	0.50%	1.00%	0.50%	2.75%	4.25%	0.00%	0.00%	0.00%	0.38%	0.60%	0.12%	0.91%
SGR	0.00%	0.17%	0.17%	0.25%	0.00%	0.25%	0.00%	0.00%	0.00%	0.00%	0.04%	0.74%	0.13%

**Table 2:** Results of SGR with and without MSRCR for bright or dark environments, where the alpha is the contrast, and the beta is brightness. (a) where alpha = 1 and beta = −100. (b) where alpha = 1 and beta = −150. (c) where alpha = 1 and beta = 75. (d) where alpha = 1 and beta = 100.

Clips/Settings		Video 1			Video 2			Video 3			Video 4		
		1-1	1-2	1-3	2-1	2-2	2-3	3-1	3-2	3-3	4-1	4-2	4-3
(a)	Original	0.00%	0.00%	0.17%	0.50%	0.50%	0.00%	0.40%	0.40%	0.40%	1.12%	1.10%	1.40%
	MSRCR	0.33%	0.00%	0.17%	0.50%	0.75%	0.00%	0.00%	0.40%	0.40%	0.00%	0.00%	1.40%
(b)	Original	0.00%	0.00%	alarm	0.50%	0.75%	0.00%	0.00%	0.00%	0.00%	0.00%	0.00%	1.40%
	MSRCR	0.00%	1.17%	0.83%	0.00%	0.75%	0.00%	0.00%	0.00%	0.40%	0.00%	1.10%	1.40%
(c)	Original	0.00%	0.33%	0.17%	0.00%	0.50%	0.00%	0.00%	0.00%	0.00%	alarm	alarm	alarm
	MSRCR	0.00%	0.00%	0.17%	0.00%	0.75%	0.00%	0.00%	0.40%	0.00%	0.00%	alarm	1.40%
(d)	Original	0.00%	0.00%	1.17%	alarm	0.50%	alarm	1.20%	0.40%	0.40%	alarm	alarm	alarm
	MSRCR	0.00%	0.00%	0.50%	0.00%	1.25%	alarm	0.80%	0.40%	0.40%	0.00%	alarm	alarm

**Table 3:** Results of SGR with and without MSRCR for a single light source environment, where the sigma is the adjustment of the exposure range. (a), (b), and (c) are a single light source illuminated at the center of image, and their sigma values are 100, 75, and 50, respectively. (d), (e), and (f) are a single light source illuminated at the left top of image, and their sigma values are 300, 200, and 100, respectively.

Clips/Settings		Video 1			Video 2			Video 3			Video 4		
		1-1	1-2	1-3	2-1	2-2	2-3	3-1	3-2	3-3	4-1	4-2	4-3
(a)	Original	alarm	alarm	alarm	alarm	1.25%	0.00%	0.40%	0.40%	0.40%	1.12%	0.00%	1.40%
	MSRCR	0.00%	0.00%	0.17%	0.00%	0.50%	0.25%	0.40%	0.40%	0.40%	0.00%	1.10%	1.40%
(b)	Original	alarm	alarm	alarm	alarm	alarm	1.50%	2.00%	alarm	alarm	alarm	alarm	alarm
	MSRCR	0.67%	alarm	alarm	0.75%	0.50%	0.25%	0.40%	0.40%	0.40%	1.12%	0.00%	1.40%
(c)	Original	alarm	alarm	alarm	alarm	alarm	alarm	alarm	alarm	alarm	alarm	alarm	alarm
	MSRCR	alarm	alarm	alarm	alarm	alarm	alarm	1.20%	1.60%	alarm	alarm	alarm	alarm
(d)	Original	0.00%	0.33%	alarm	0.50%	0.50%	0.25%	0.40%	0.40%	0.00%	0.00%	0.00%	1.40%
	MSRCR	0.33%	0.00%	0.17%	0.50%	0.50%	0.00%	0.40%	0.40%	0.40%	0.00%	0.00%	1.40%
(e)	Original	alarm	alarm	alarm	1.75%	0.50%	0.25%	0.00%	0.00%	0.00%	alarm	alarm	alarm
	MSRCR	0.00%	0.00%	0.50%	1.75%	0.75%	0.00%	0.00%	0.80%	0.00%	1.12%	1.10%	1.40%
(f)	Original	alarm	alarm	alarm	alarm	alarm	alarm	alarm	alarm	alarm	alarm	alarm	alarm
	MSRCR	alarm	alarm	alarm	alarm	alarm	alarm	alarm	0.00%	1.20%	alarm	alarm	alarm
